# Diagnostic accuracy and clinical value of [68Ga]Ga-FAPI-46 PET/CT for staging patients with ovarian cancer: study protocol for a prospective clinical trial

**DOI:** 10.1186/s12885-024-12461-w

**Published:** 2024-06-07

**Authors:** Morten Bentestuen, Morten Ladekarl, Aage Knudsen, Helle D. Zacho

**Affiliations:** 1 Department of Nuclear Medicine and Clinical Cancer Research Center, Aalborg University Hospital, Hobrovej 18- 22, Aalborg, DK-9000 Denmark; 2 Department of Oncology and Clinical Cancer Research Center, Aalborg University Hospital, Hobrovej 18-22, Aalborg, DK- 9000 Denmark; 3 Department of Gynecology and Obstetrics, Aalborg University Hospital, Reberbansgade 15, Aalborg, DK-9000 Denmark; 4 Department of Clinical Medicine, Aalborg University, Sdr. Skovvej 11, Aalborg, DK-9000 Denmark

**Keywords:** [^68^Ga]Ga-FAPI-46, FAPI, Fibroblast activation protein inhibitor, PET, Positron emission tomography, Ovarian cancer

## Abstract

**Background:**

[^18^F]Fluorodeoxyglucose ([^18^F]FDG) positron emission tomography (PET) is recommended during diagnostic work-up for ovarian cancer; however, [^18^F]FDG PET has several inherent limitations. The novel oncologic PET-tracer fibroblast activation protein inhibitor (FAPI) has demonstrated promising results in multiple cancer types, including ovarian cancer, and could overcome the limitations of [^18^F]FDG PET; however, high-quality clinical studies are lacking. The primary objective of the present study is to compare the diagnostic accuracy of [^68^Ga]Ga-FAPI-46 PET/CT and [^18^F]FDG PET/CT in ovarian cancer patients and to investigate how this potential difference impacts staging and patient management.

**Methods and design:**

Fifty consecutive ovarian cancer patients will be recruited from Aalborg University Hospital, Denmark. This study will be a single-center, prospective, exploratory clinical trial that adheres to the standards for reporting diagnostic accuracy studies (STARD). This study will be conducted under continuous Good Clinical Practice monitoring. The eligibility criteria for patients are as follows: (1) biopsy verified newly diagnosed ovarian cancer *or* a high risk of ovarian cancer *and* referred for primary staging with [^18^F]FDG PET/CT; and (2) resectable disease, i.e., candidate for primary debulking surgery *or* neoadjuvant chemotherapy followed by interval debulking surgery. All recruited study subjects will undergo [^68^Ga]Ga-FAPI-46 PET/CT at primary staging, before primary debulking surgery or neoadjuvant chemotherapy (Group A + B), in addition to conventional imaging (including [^18^F]FDG PET/CT). Study subjects in Group B will undergo an additional [^68^Ga]Ga-FAPI-46 PET/CT following neoadjuvant chemotherapy prior to interval debulking surgery. The results of the study-related [^68^Ga]Ga-FAPI-46 PET/CTs will be blinded, and treatment allocation will be based on common clinical practice in accordance with current guidelines. The histopathology of surgical specimens will serve as a reference standard. A recruitment period of 2 years is estimated; the trial is currently recruiting.

**Discussion:**

To our knowledge, this trial represents the largest, most extensive, and most meticulous prospective FAPI PET study conducted in patients with ovarian cancer thus far. This study aims to obtain a reliable estimation of the diagnostic accuracy of [^68^Ga]Ga-FAPI-46 PET/CT, shed light on the clinical importance of [^68^Ga]Ga-FAPI-46 PET/CT, and examine the potential applicability of [^68^Ga]Ga-FAPI-46 PET/CT for evaluating chemotherapy response.

**Trial registration:**

clinicaltrials.gov: NCT05903807, 2nd June 2023; and euclinicaltrials.eu EU CT Number: 2023-505938-98-00, authorized 11th September 2023.

## Background

In Europe, ovarian cancer (OC) ranks as the fifth leading cause of death among female cancer-related fatalities. In 2020, 27,138 new cases were reported, accounting for 4.9% of all female cancer deaths [1]. Seventy-to-80% of all OC patients are diagnosed at advanced stages because symptoms are discrete and often caused by peritoneal metastases [2]. Early detection and complete cytoreduction (no visible disease, R0 resection) or optimal cytoreduction (leaving tumor nodules < 1 cm, R1 resection) are crucial for treatment and survival [3, 4]. Treatment for OC involves curatively intended primary debulking surgery (PDS) followed by adjuvant systemic chemotherapy; however, in advanced stages where complete cytoreduction by PDS is unlikely, interval debulking surgery (IDS) after 2–3 cycles of neoadjuvant chemotherapy (NACT) is the preferred treatment option [5].

Imaging modalities play a crucial role in the clinical work-up of OC patients and are fundamental for efficient diagnosis and correct patient management. In summary, ultrasound (US) is essential for screening and diagnosis, while magnetic resonance imaging (MRI), computer tomography (CT), and [^18^F]fluorodeoxyglucose positron emission tomography ([^18^F]FDG PET) are useful in evaluating the extent of disease [6–9]. Moreover, multiple imaging-based models have been proposed for the preoperative prediction of cytoreducibility. Although these models provide clinically useful information, no single imaging-based prediction model is adequate for accurately predicting the outcome of a surgical procedure [10–13].

[^18^F]FDG PET/CT, an imaging modality visualizing elevated glycolysis, is a highly valuable tool in OC, as it can improve the efficacy of OC diagnosis, recurrence detection, staging and prognosis. In particular, this imaging modality has high sensitivity for detecting lymph node metastases and extra-abdominal metastases [14–21]. However, [^18^F]FDG PET has several limitations. First, [^18^F]FDG is suboptimal for discerning borderline tumors from benign tumors [16, 17]. Second, [^18^F]FDG uptake is low in certain histological subtypes, i.e., mucinous, clear-cell and cystic carcinomas [22]. Third, false-positive [^18^F]FDG PET uptake in ovaries can be observed in several benign ovarian conditions [23, 24]. Moreover, many benign conditions can be misinterpreted as peritoneal or distant organ metastases [21, 25]. Fourth, the predictive value of [^18^F]FDG PET for determining the likelihood of achieving complete cytoreduction is limited [20, 26–28]. Also, malignant peritoneal lesions demonstrate varying [^18^F]FDG uptake, leading to suboptimal performance in predicting peritoneal carcinomatosis and/or predicting the outcome of surgery [13, 29].

[^18^F]FDG PET imaging plays a crucial role in the diagnostic evaluation of OC and is recommended in guidelines [5, 30]; however, to optimize the diagnostic performance of PET in OC, it is necessary to overcome the inherent limitations of the tracer [^18^F]FDG.

Fibroblast activation protein (FAP) is a transmembrane protein that can be upregulated in solid tumors, including epithelial carcinomas [31–33]. In most tumors in which FAP is present, FAP is not directly expressed by the cancer cells themselves but by cancer-associated fibroblasts (CAFs), which represent a major part of the gross tumor volume. In 2018, the first results of [^68^Ga]Ga-FAPI PET/CT were published, with promising results both in animal cancer models and in cancer patients [32, 34, 35]. In pathological studies of human cancer tissue, FAP expression has been detected in 85–97% of ovarian cancers, both in serous and mucous subtypes. Moreover, FAP is absent/low in normal ovarian tissue and under benign conditions [33, 36–38]. Immediately after the development of the FAPI, the clinical value of FAPI PET/CT in OC patients was highlighted by several case reports in which FAPI PET/CT detected far more peritoneal metastases than [^18^F]FDG PET/CT [39–41]. Additionally, FAPI uptake in ovaries does not seem to be affected by the menstrual cycle, as is the case with [^18^F]-FDG [42, 43].

Only recently have cohort studies been conducted on ovarian cancer. In a retrospective study of nonconsecutive patients, FAPI PET/CT outperformed FDG PET/CT in terms of sensitivity for primary tumors (100% vs. 78%), lymph nodes (100% vs. 80%) and peritoneal metastases (100% vs. 56%). In this study, 36% of newly diagnosed ovarian cancer patients were correctly upstaged on FAPI PET/CT due to missed lesions on [^18^F]FDG PET/CT [44]. Another retrospective study confirmed that, compared with FDG PET/CT, [^68^Ga]Ga-FAPI-04 PET/CT correctly detected twice the number of peritoneal metastases [45]. In a study of patients with suspected relapse of OC, [^68^Ga]Ga-FAPI-04 PET/CT outperformed [^18^F]FDG PET/CT (overall pooled lesion sensitivity: 96% vs. 49%), and the chosen treatment was altered in 17% of the patients [46]. In a prospective study by Chen et al., [^68^Ga]Ga-FAPI-04 PET/CT and [^18^F]FDG PET/CT demonstrated equivalent sensitivities for primary tumors (approximately 92–94%) but significantly greater sensitivities for [^68^Ga]Ga-FAPI-04 PET when assessing lymph node metastases and peritoneal metastases (81% vs. 61% and 98% vs. 76%, respectively), leading to upstaging in 14% of treatment-naïve patients and 33% of relapsed patients and a change in treatment in 11% of treatment-naïve patients and 19% of relapsed patients [47]. However, according to the results of another prospective study, [^68^Ga]Ga-FAPI PET/CT and [^18^F]FDG PET/CT demonstrated comparable sensitivities for primary tumor and lymph node metastases (approximately 90–100% for both tracers), and [^68^Ga]Ga-FAPI PET/CT had only slightly better sensitivity for peritoneal lesions [48].

Although most reports on FAPI PET in patients with ovarian cancer seem convincing, there are several limitations, including relatively small cohorts and mostly retrospective study designs. The few prospective studies performed do not include consecutive patients, and most findings (beyond the primary tumor) are not comparable to a histopathological reference standard. Moreover, the effect of chemotherapy on FAPI uptake and the feasibility of using FAPI PET/CT for evaluating patient response after NACT are unknown. These latter concepts are highly relevant because approximately 75% of OC patients are diagnosed at advanced stages and undergo interval cytoreductive surgery following neoadjuvant chemotherapy [49]. Studies overcoming these limitations are therefore needed before randomized trials using FAPI PET/CT can be designed to implement FAPI PET in clinical practice for the potential benefit of future cancer patients.

By conducting the present comparative diagnostic accuracy study of [^68^Ga]Ga-FAPI-46 PET/CT and [^18^F]-FDG PET/CT in OC, we aim to answer several of these questions, providing a significant contribution to the rapidly evolving field of diagnostic [^68^Ga]Ga-FAPI-46 PET in OC and other cancer entities.

## Methods and design

### Study characteristics and identification

The present study will be a single-center, prospective, explorative clinical trial investigating the diagnostic accuracy and clinical value of the novel, oncologic, diagnostic PET tracer [^68^Ga]Ga-FAPI-46 in patients with newly diagnosed ovarian cancer. The study protocol has been uploaded to clinicaltrials.gov (NCT05903807) and euclinicaltrials.eu (EU CT: 2023-505938-98-00. The study will take place in its entirety at Aalborg University Hospital (Aalborg UH), Denmark.

### Study objectives

#### Primary objectives

The primary objectives are to compare the FAPI PET/CT and FDG PET/CT findings in primary tumors, regional lymph nodes and distant metastases to a histopathological reference standard to the sensitivity, specificity, positive predictive value (PPV), and negative predictive value (NPV) of the PET/CTs, both at primary staging and post-NACT staging (i.e., staging after NACT, prior to interval debulking). This will include a comparison of the cancer stage determined by FAPI PET/CT and conventional imaging (including [^18^F]FDG PET/CT) at primary staging and post-NACT staging [50, 51]. The proportions of patients downstaged, having unchanged stage, and being upstaged according to the added FAPI PET/CT will be determined.

### Secondary objectives

The secondary objectives are as follows: (1) To estimate the proportion of patients hypothetically treated differently due to [^68^Ga]Ga-FAPI-46 PET/CT replacing [^18^F]FDG PET/CT; (2) to investigate the intensity of [^68^Ga]Ga-FAPI-46 uptake in tumor deposits; (3) to investigate the feasibility of FAPI PET/CT for chemotherapy-response assessment compared to FDG PET/CT; (4) to investigate the prognostic value of FAPI PET/CT compared to FDG PET/CT at primary staging and post-NACT staging; (5) to evaluate potential unexpected FAPI PET/CT findings; and (6) to evaluate the safety of [^68^Ga]Ga-FAPI-46 injections.

### Study design

#### Study flow overview

This prospective study will comply with the Standard for Reporting Diagnostic Accuracy (STARD) criteria. Good Clinical Practice (GCP) will be followed to the extent that this is a diagnostic test accuracy trial involving an investigational medicinal product (IMP) [52].

**Fig. 1:**
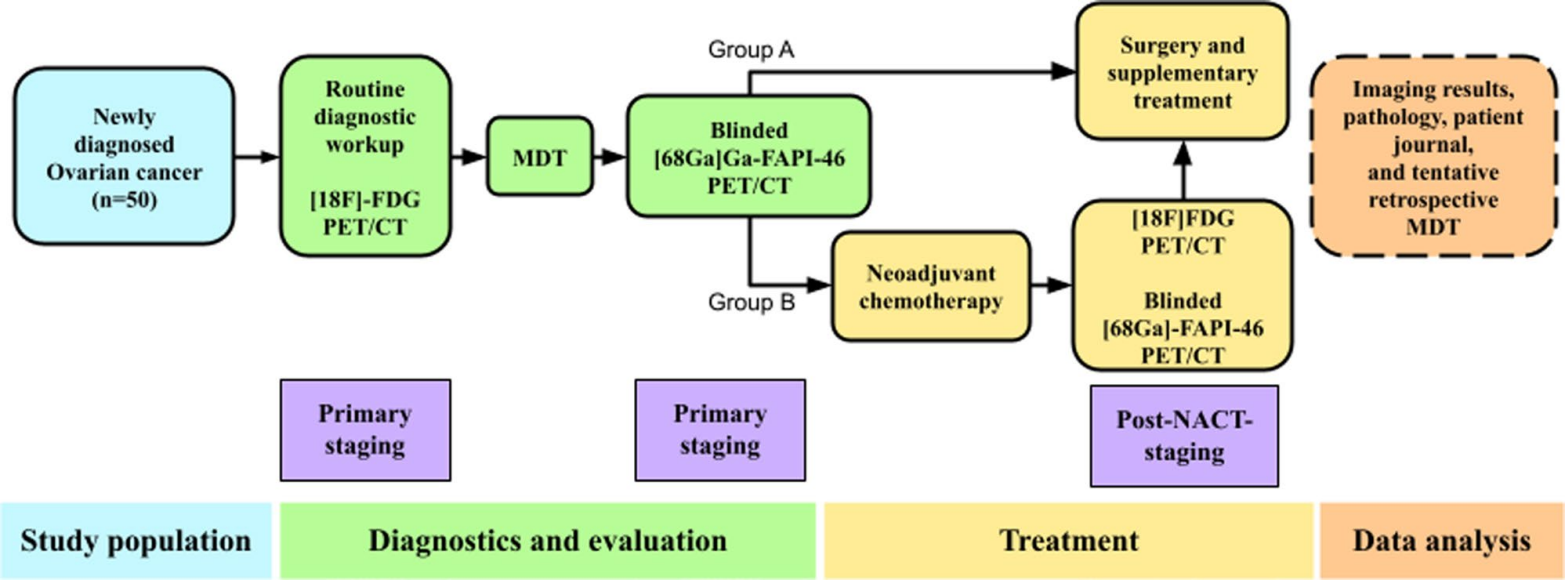
FAPI PET in ovarian cancer study design and flow

Patients with newly diagnosed biopsy-verified OC *or* highly suspected OC (based on all the information presented at the Gynecological Cancer Multidisciplinary Team Conference (MDT)) with operable and resectable disease will be identified at the MDT. Based on the standard diagnostic workup, which includes [^18^F]FDG PET/CT and a diagnostic contrast enhanced CT (ceCT), and in line with national and international guidelines, patients will either go directly to surgery (Group A) or undergo neoadjuvant chemotherapy prior to surgery (Group B). Patients in both groups (A + B) will undergo FAPI PET/CT prior to treatment and within 1 week of receiving the primary FDG PET/CT (Fig. 1). The additional FAPI PET/CT will not interfere with or delay routine diagnostic workup or treatment, and the results of the FAPI PET/CT will not be available to the patient or the treating physician. Patients in Group B will undergo post-NACT-staging with conventional imaging (including [^18^F]FDG PET/CT) 2–3 weeks after NACT. In addition to conventional imaging, an additional blinded FAPI PET/CT will be performed at post-NACT staging.

[^68^Ga]-Ga-FAPI-46 PET/CT data will be interpreted after enrollment of all patients and compared to the [^18^F]-FDG PET/CT data. Histopathology of the resected specimens and biopsies will serve as a reference standard for calculating the diagnostic accuracy. For suspicious lesions not confirmed by histopathology, other imaging modalities (US, MRI, CT), biochemistry, and medical records will be used for classification. The clinical impact of [^68^Ga]Ga-FAPI-46 PET/CT, both at primary staging and post-NACT staging, will be evaluated at a retrospective tentative MDT with the participation of an onco-gynecological surgeon, an oncologist, a pathologist, and a nuclear medicine physician, all of which specialize in the treatment of ovarian cancer. The tentative MDT will be performed retrospectively and therefore has no influence on the actual treatment of patients; moreover, the MDT differs from the clinical MDT, as clinicians will present study-related [^68^Ga]Ga-FAPI-46 PET/CTs instead of [^18^F]FDG PET/CTs. The study flow is depicted in Fig. 1.

### Study population

#### Sample size and patients

Patients with biopsy-verified OC prior to the gynecological cancer MDT *or* highly suspected to have OC (based on all information presented at the gynecological cancer MDT) will be recruited until a total of 50 patients with histopathologically confirmed OC have been included. The upper limit of recruited patients is set at 60. Recruitment will take place at an outpatient clinic related to the Dept. of Gynecology and Obstetrics Aalborg University Hospital (Aalborg UH) by trained staff in accordance with national requirements [53]. Because an official sample size calculation is not feasible due to former study limitations, e.g., highly selected patients and lack of histopathology of metastases, the desired number of participants is based on the possibility of recruiting patients within 1.5-2 years at our institution, and more importantly, valid estimates of diagnostic accuracy are obtainable with the planned number of patients in an unselected consecutive cohort. Approximately 25 patients are estimated to undergo direct surgery after [^68^Ga]Ga-FAPI-46 PET/CT (Group A), and 25 patients will undergo NACT followed by a second [^68^Ga]Ga-FAPI-46 PET/CT (Group B). The recruitment strategy is based on unpublished clinical data from our institution and from published literature, where approximately 10–15% of the patients with highly suspected OC (based on all available information at MDT) had other non-OC malignancies or rarely benign conditions upon histopathology, e.g., endometriosis, gastric cancer (i.e., Krukenberg tumors), and colorectal cancer [24, 54].

### Study participant eligibility

The inclusion criteria are; (1) Patients newly diagnosed with biopsy-verified OC *or* highly *s*uspected to have OC (based on all the data presented at the gynecological cancer MDT) *and* referred to primary staging with [^18^F]FDG PET/CT; (2) patients deemed resectable and operable at the MDT with or without neoadjuvant chemotherapy; (3) patients considered physically and mentally able to participate in the research project; (4) patients eighteen years or older and able to consent to participate in the project; and (5) patients can read and understand Danish. The main exclusion criteria are: (1) Patients unfit for surgery; (2) patients with recurrent OC; (3) patients with concurrent non-OC malignancies; (4) patients unable to participate in PET/CT; (5) patients with a history of hypersensitivity to [^18^F]FDG or FAPI tracers; (6) pregnant, lactating, or breastfeeding patients or fertile women not using effective contraceptives; and (7) known medical conditions that would significantly decrease the reliability of the data obtained as part of this study. A full set of exclusion criteria can be found at clinicaltrials.gov (NCT05903807) and euclinicaltrials.eu (EU CT: 2023-505938-98-00).

### Imaging procedure

A dose of 200 *±* 50 Megabecquerel (MBq) [^68^Ga]Ga-FAPI-46 will be injected through an antecubital peripheral venous catheter. Patients will be encouraged to per-oral intake of water and instructed to empty their bladder immediately before PET/CT acquisition, which will be performed 30 *±* 5 min after tracer injection—no other patient preparation is needed. Before imaging, a scout scan will be performed to determine the scan area, which will be identical to the [^18^F]FDG PET/CT scan area. First, a low-dose CT scan (2–4 mSv) will be performed immediately followed by PET image acquisition. The scan procedure will last for approximately 25 min, after which the patients can immediately go home. Presently, there are no guidelines on how to perform a FAPI PET. The FAPI PET procedure used in the present study protocol is based on the best available evidence in the published literature and a systematic review conducted by the investigator [55]. The [^18^F]FDG PET/CT scan procedure will be performed in accordance with the dept. of Nuclear Medicines, Aalborg UHs local instructions and international guidelines [56]. Diagnostic ceCT covering the entire PET field-of-view is conducted as part of common clinical practice, both at primary staging and at post-NACT staging, and will be available when interpreting the PET scans.

### Study-related procedures and data analysis

#### Pathology analysis

The clinical trial will not influence the amount of biological material collected from the patients. The surgical specimens will be divided into different containers during surgery and subsequently sent to the Dept. of Pathology, with labels indicating the exact location according to common clinical practice, enabling correct correlation of the histopathology of lesions to the imaging modalities. All surgical specimens will undergo histopathological examination according to routine clinical practice, with additional analyses, including quantitative evaluations of the density and intensity of FAP-expressing tumor cells or CAFs by applying immunohistochemistry. Standardized uptake values (SUVs) in tumor deposits will be compared to tumor characteristics, as determined by pathology, including immunohistochemistry.

### Diagnostic accuracy

[^68^Ga]Ga-FAPI-46 PET/CT images will be independently interpreted under standardized conditions by two experienced nuclear medicine physicians, considering the currently known pitfalls in FAPI imaging [57–59]. The interpreters will be blinded to all clinical information regarding the patients except for the cancer diagnosis. Any disagreements that arise will be resolved through a consensus-based approach. Likewise, the [^18^F]FDG PET/CTs will be interpreted under standardized condition by experienced nuclear medicine physicians. ceCTs performed at primary staging and at post-NACT staging is part of the PET/CT procedure and will also be available for the interpreters and be used for deeming malignancy in suspicious lesions with low [^18^F]FDG / [^68^Ga]Ga-FAPI-46 uptake.

The correlations of the PET/CT results with histopathology, estimated sensitivities, specificity, PPV, and NPV for [^68^Ga]Ga-FAPI-46 PET/CT and [^18^F]FDG PET/CT findings will be compared for primary tumors, lymph nodes and distant metastases (e.g., peritoneal metastases). Ultimately, the diagnostic accuracy of [^68^Ga]Ga-FAPI-46 PET/CT and [^18^F]FDG PET/CT will be compared at primary staging and post-NACT-staging. Suspicious tumor lesions revealed on [^68^Ga]Ga-FAPI-46 PET/CT lacking histopathological confirmation will be evaluated using available routine diagnostic images ([^18^F]FDG PET/CT, UL, MR, ceCT), medical records, and laboratory tests. Furthermore, correlating [^68Ga^]Ga-FAPI-46 PET/CT at post-NACT staging with histopathology will be useful for assessing the feasibility of [^68^Ga]Ga-FAPI-46 PET/CT after chemotherapy.

### Staging and impact on patient management

Tentative staging based on FAPI and [^18^F]FDG PET/CT data will be performed according to the International Federation of Gynecological Oncology’s classification system (FIGO) and the American Joint Committee on Cancer (AJCC 8th) edition TNM classification. Staging, as determined at the tentative MDT, will be compared for [^68^Ga]Ga-FAPI-46 PET/CT and [^18^F]FDG PET/CT, both at primary staging and post-NACT staging, and patients will be classified as “upstaged”, “unchanged”, or “downstaged” due to [^68^Ga]Ga-FAPI-46 PET/CT. The proportions of patients in these categories will be calculated. Furthermore, at the tentative MDT, the treatment strategy (e.g., operability, resectability, palliative treatment, other treatment) will be evaluated purely based on the [^68^Ga]Ga-FAPI-46 PET/CTs (replacing [^18^F]FDG PET/CTs) both at primary staging and at post-NACT staging. The proportion of patients with a hypothetically changed treatment strategy will be calculated. Supplementary imaging results (excluding the FDG PET/CT results), biochemical data, and medical records prior to the staging time point will be available for the clinicians attending the tentative MDT to mimic the situation at the clinical MDT.

### Uptake parameters and chemotherapy evaluation

The SUV and tumor-to-background ratio (TBR) for the primary tumor, lymph nodes and distant metastases will be calculated for [^68^Ga]Ga-FAPI-46 and [^18^F]FDG PET/CT. Furthermore, the total volume of pathological [^68^Ga]Ga-FAPI-46 and [^18^F]FDG uptake (molecular imaging tumor volume for [^68^Ga]Ga-FAPI-46 PET and [^18^F]FDG PET [MITV_FAPI_ and MITV_FDG]_) and total lesion uptake on [^68^Ga]Ga-FAPI-46 PET and [^18^F]FDG PET (volume intensity product VIP for [^68^Ga]Ga-FAPI-46 [VIP_FAPI_ and VIP_FDG]_)) will be calculated, both at primary staging and at post-NACT staging. These uptake parameters will be compared between [^68^Ga]Ga-FAPI-46 PET/CT and [^18^F]FDG PET/CT at primary staging and post-NACT staging, and the changes in these parameters, from primary staging to post-NACT staging, will be compared with histopathology.

### Prognostic value FAPI PET/CT

Patients will be followed for up to 10 years to determine recurrence-free survival (RFS) and overall survival (OS). The prognostic value of [^68^Ga]Ga-FAPI-46 PET/CT and [^18^F]FDG PET/CT and FAP-specific pathological analyses will be assessed with multivariate analysis, including stage, tumor subtype and grade, molecular alterations, and other potential prognostic parameters.

### Safety evaluation

The reported heart rate, blood pressure and reported discomfort will be evaluated at baseline, 1 min post-injection, 10 min post-injection, and after [^68^Ga]Ga-FAPI-46 PET/CT (approximately 60 min post-injection), providing the first systematic safety evaluation of [^68^Ga]Ga-FAPI-46.

### Unexpected and non-OC conditions

The nature of unexpected [^68^Ga]Ga-FAPI-46 PET/CT findings will be evaluated by seeking information in the patient’s medical records, imaging modalities, laboratory tests, and pathology. Moreover, the FAPI PET/CT data of patients with revealed “non-OC conditions” upon histopathology, will also be analyzed for the purpose of assessing FAPI PET/CT under these conditions.

### Ethical considerations

Study subjects will undergo 1 or 2 [^68^Ga]Ga-FAPI-PET/CTs in Group A or Group B, respectively, with an estimated additional radiation dose of 3.2–6.0 mSv per [^68^Ga]Ga-FAPI-46 PET/CT [60]. Converted to a lifetime risk of incurable cancer, the risk increases by 0.03% in study Group A and 0.06% in study Group B (higher estimates) [61]. These estimates are based on a linear model for higher radiation doses in a younger study population and are therefore likely exaggerated. There will be no immediate advantages of participation in the study. As the clinical value of [^68^Ga]Ga-FAPI-PET in OC is unknown, the results of study-related [^68^Ga]Ga-FAPI-46 PET/CTs will not be revealed to the patient or the treating physician. The study has been designed to be of the least inconvenience for the participants, and participants can withdraw their consent at any time without consequences.

## Discussion

Early diagnosis and correct evaluation of the extent of disease are crucial for optimal management of patients with OC. US, CT, MRI and [^18^F]FDG PET/CT are presently recommended for diagnostic workup; however, these imaging modalities have limitations, especially regarding the relatively low sensitivity for detecting peritoneal metastases, thereby limiting their ability to predict incomplete cytoreduction. The novel PET tracer [^68^Ga]Ga-FAPI-46 has shown promising results in former studies, including those involving OC patients and patients with peritoneal carcinomatosis, but these studies have several limitations. In the present study, we aim to provide a valid estimate of the diagnostic accuracy of [^68^Ga]Ga-FAPI-46 for OC at primary staging and post-NACT staging and to investigate the potential influence of [^68^Ga]Ga-FAPI-46 PET/CT on patient management.

The Grading of Recommendations, Assessments, Development and Evaluation (GRADE) guidelines state that valid diagnostic test accuracy studies should include representative and consecutive cohorts [62]. In our study, consecutive patients with newly diagnosed biopsy-verified OC or a high risk of OC will be included. Although this design does not enable the inclusion of all patients admitted to a specialized center for OC, the design was chosen to optimize the likelihood of revealed OC upon histopathology, thereby limiting the dropout of patients with revealed non-OC conditions. Furthermore, the patient cohort will not include a small group of patients diagnosed with very limited stage disease. The STARD guidelines for reporting diagnostic test accuracy are endorsed by the Cochrane organization and will be followed in the present study when manuscripts are produced [52]. Conducting studies in accordance with the STARD criteria is crucial for providing comparable data, enabling future meta-analyses, and randomized controlled trials. Adherence to these recommendations and guidelines makes our study the most thorough study to date.

We designed this study to evaluate the potential clinical role and value of [^68^Ga]Ga-FAPI-46 PET/CT because these aspects have been understudied in previous reports. For this purpose, the study will be conducted in a clinical hospital setting with minimal alterations to routine diagnostics, and [^68^Ga]Ga-FAPI-46 PET/CT-based tentative staging and hypothetical alterations to patient management will be evaluated and compared to conventional imaging modalities, including [^18^F]FDG PET/CT, both at primary staging and post-NACT staging. Moreover, as the prognostic value of [^68^Ga]Ga-FAPI-46 uptake parameters (i.e., SUV, TBR, MITV, and VIP) in tumor lesions at primary staging and after NACT is presently unknown, our clinical trial included follow-up data according to recurrence-free survival and overall survival. These data could be informative in discerning responders from nonresponders and for assessing the probability of cure.

In the present study, primary and post-NACT-staging with [^18^F]FDG PET/CT and [^68^Ga]Ga-FAPI-46 PET/CT will be conducted. This allows us to explore the feasibility/reliability of [^68^Ga]Ga-FAPI-46 PET imaging after chemotherapy. Moreover, changes in uptake parameters may aid in discerning patients who respond to chemotherapy from nonresponders. Investigating [^68^Ga]Ga-FAPI-46 uptake parameters and the impact of chemotherapy on these parameters are of particular interest because FAPI-46 has potential for radionuclide therapy, a field that is expected to rapidly evolve in the immediate future [63].

## Data Availability

No datasets were generated or analysed during the current study.

## Declarations

### Trial status

Currently recruiting (start of recruitment December 2023). Estimated end of recruitment: December 2025.
